# Re-analysis of a Genome-Wide Gene-By-Environment Interaction Study of Case Parent Trios, Adjusted for Population Stratification

**DOI:** 10.3389/fgene.2020.600232

**Published:** 2021-01-13

**Authors:** Pulindu Ratnasekera, Brad McNeney

**Affiliations:** Department of Statistics and Actuarial Science, Simon Fraser University, Burnaby, BC, Canada

**Keywords:** gene-environment interaction, case-parent trios, population stratification, genome-wide association study, cleft palate

## Abstract

We investigate the impact of confounding on the results of a genome-wide association analysis by Beaty et al., which identified multiple single nucleotide polymorphisms that appeared to modify the effect of maternal smoking, alcohol consumption, or multivitamin supplementation on risk of cleft palate. The study sample of case-parent trios was primarily of European and East Asian ancestry, and the distribution of all three exposures differed by ancestral group. Such differences raise the possibility that confounders, rather than the exposures, are the risk modifiers and hence that the inference of gene-environment (*G*×*E*) interaction may be spurious. Our analyses generally confirmed the result of Beaty et al. and suggest the interaction *G*×*E* is driven by the European trios, whereas the East Asian trios were less informative.

## 1. Introduction

In a case-parent trio study we collect genotypes, *G*, on affected children and their parents. We may also collect environmental exposures or non-genetic attributes, *E*, on the children. Gene-environment interaction (*G* × *E*) is a statistical interaction term in the standard log-linear model of association between covariates and disease status. *G* × *E* interaction can be interpreted as genes that modify the effect of an exposure, or as an exposure that modifies the effect of genes.

Beaty et al. (2011) conducted an analysis of the GENEVA Oral Cleft Study data, a genome-wide association study identifying genetic and environmental factors associated with cleft palate (CP). They found multiple single nucleotide polymorphisms (SNPs) appeared to modify the effect of maternal smoking, maternal alcohol consumption, or maternal multivitamin supplementation on the risk of CP. The study sample of case-parent trios was primarily of European and East Asian ancestry, and the distribution of all three exposures differ by ancestral group, which raises the possibility of spurious *G* × *E*.

When the test locus, *G*′, is not causal, disease risk at *G*′ can appear to be modified by *E* without *G* × *E* interaction when there is exposure-related population structure (Shi et al., 2011; Weinberg et al., 2011). Exposure-related population structure may be thought of as a form of confounding that occurs when both *GG*′ haplotype frequencies and *E* distributions differ by ancestral group. Differences in *GG*′ haplotypes can lead to differences in *G*′ risk that may be tagged by *E*, suggesting *G*′ × *E* even in the absence of true *G* × *E* interaction.

One of the requirements for exposure-related population structure is different *E* distributions in the different ancestral groups. Such is the case in the GENEVA Oral Cleft Study where maternal smoking, maternal alcohol consumption and maternal multivitamin supplementation were all more common in self-reported Europeans than in self-reported East Asian populations (see **Table 2**). If, in addition, haplotype frequencies for markers in the vicinity of a causal SNP also vary by ancestral groups, any inferences of *G* × *E* interaction could be spurious. Shin et al. (2012) proposed bias-reduced methods for inference of *G* × *E* interaction from case-parent trio data in the presence of exposure-related population structure. In this article we use these methods to adjust the analyses of Beaty et al. (2011) for potential confounding effects of population stratification.

## 2. The GENEVA Oral Cleft Study

The GENEVA Oral Cleft study (gen, 2010) was comprised of 550 case-parent trios from 13 different sites across the United States, Europe, Southeast Asia, and East Asia. For our analyses, data were obtained through dbGAP at https://www.ncbi.nlm.nih.gov/projects/gap/cgi-bin/study.cgi?study_id=phs000094.v1.p1 with accession number phs000094.v1.p1. Of the 550 trios included in the GENEVA Oral Cleft study, 462 were available for our analyses. Summaries of the trios by ancestry and gender of the affected child are shown in Table 1.

**Table 1:** Gender of 462 CP cases in the international consortium study by ancestral group.

**Ancestry group**	**Males**	**Females**	**Total**	**%**
European	103	111	214	46
Asian	93	141	234	51
Other/Afr	3	11	14	3
Total	199	263	462	100

The objective of the GENEVA Oral Clefts study was to discover genetic contributions to orofacial clefts, the most common type of craniofacial birth defect in humans, and to assess whether these genes modify the effect of exposures known to be associated with cleft palate. Maternal exposure to multivitamins, alcohol and smoking were assessed through maternal interviews focused on the peri-conceptual period (3 months prior to conception through the first trimester), which includes the first 8–9 weeks of gestation when palatal development is completed. Exposure status is summarized in Table 2. From this table we see the ancestry of the sample is predominantly European (46%) and East Asian (51%), and these three exposures are all more common in Europeans.

**Table 2:** Exposure rates for maternal alcohol consumption, maternal smoking, and maternal vitamin supplementation for the total CP group and for three ancestral groups.

	**Exposure (%) to maternal**			
**Ancestry group**	**Alcohol consumption (%)**	**Smoking (%)**	**Vitamin supplementation (%)**	**Affected children**
European	41	28	57	214
East Asian	4	3	21	234
Other/Afr	14	7	71	14
Total	21	14	39	462

Beaty et al. found evidence for *G* × *E* interaction for maternal alcohol consumption and SNPs in the genes *MLLT3* and *SMC2*, for maternal smoking and SNPs in the genes *TBK1* and *ZNF236*, and for maternal multivitamin use and SNPs in the *BAALC* gene.

## 3. Models and Methods

*G* × *E* interaction is defined as the statistical interaction term β_*GE*_ in a log-linear model for the probability of disease:

(1) $$\begin{array}{l} \log\left[P\left(D=1|G=g,E=e\right)\right]=\beta_{0}+g\beta_{G}+e\beta_{E}+ge\beta_{GE}, \end{array}$$

where *D* = 1 indicates the child is affected with cleft palate, *G* is the child's genotype, coded as 0, 1, or 2 copies of the minor allele, *E* is an exposure variable and β_*E*_ is the environmental main effect, which cannot be directly estimated in the case-parent trio design. To simplify the presentation, we assume throughout *E* is a binary variable with value 1 indicating exposure and 0 indicating no exposure. A non-zero interaction effect, β_*GE*_, suggests *G* modifies the effect of *E* on disease risk.

Assuming *G* and *E* are independent given parental genotypes *G*_*p*_, and the risk model in Equation (1), one can derive the conditional distribution of *G* given *D* = 1, *E* and *G*_*p*_, which can be stated in terms of the *genotypic odds*

$$\begin{array}{l} \frac{P\left(G=g|D=1,E=e,G_{p}=g_{p}\right)}{P\left(G=g-1|D=1,E=e,G_{p}=g_{p}\right)}=\exp\left(k_{p}+\beta_{G}+e\beta_{GE}\right) \end{array}$$

for a constant *k*_*p*_ that depends on *g*_*p*_ (Appendix A.1). The environmental main effect does not appear in this genotypic odds and cannot be estimated from case-parent trio data. In general, any regression effect that does not involve *G* cannot be estimated from case-parent trio data.

To reduce bias from population stratification, robust methods are required. Robust methods may be classified as design- or data-based. For a binary environmental exposure, the design-based approach of Shi et al. (2011) augments the basic case-parent trio with exposure information on an unaffected sibling. Shi et al. showed that by including the sibship-averaged exposure over two sibs (affected/unaffected) in the linear model controls this potential bias. Weinberg et al. (2011) showed all information about interaction in the tetrad design of Shi et al. (2011) comes from the siblings, not the parents, which lead them to propose a sibling-augmented case-only design and analysis. Shin et al. (2012) took a data-based approach and replaced the sibship-averaged exposure of Shi et al. (2011) with the predicted exposure given ancestry, as reflected by principal components (PCs) computed from independent genetic markers. Their data-based approach is applicable for arbitrary exposures, including continuous measures, and does not require additional siblings. In this report we consider this data-based approach of Shin et al. (2012) to explore whether the *G* × *E* interaction effects reported by Beaty et al. (2011) could be spurious.

Let *X*_*E*_ be a categorical variable indicating ancestral groups with different *E* distributions. Shin et al. introduce a separate genetic effect for each level of *X*_*E*_ into the risk model Equation (1). When *X*_*E*_ is binary, this modified risk model becomes:

$$\begin{array}{l} \text{log}[P(D=1|G=g,E=e,X_{E}=x)]=\beta_{0}+g\beta_{G}+e\beta_{E}+x\beta_{X_{E}}\\ \;+ge\beta_{GE}+gx\beta_{GX_{E}}+ex\beta_{EX_{E}}+gex\beta_{GEX_{E}} \end{array}$$

In the above model, β_*G*_*X*__*E*__ controls bias by allowing different genetic effects in the two *X*_*E*_-groups. The term β_*GE*_*X*__*E*__ allows for different *G* × *E* effects in the two groups, which can improve power to detect *G* × *E* interaction (Shin et al., 2012).

As *X*_*E*_ is not known, it must be replaced by some surrogate, ${\hat{X}}_{E}$. We consider two surrogates, (i) the expectation of *E* given genetic markers (EEGM) and (ii) self-reported ancestry (SRA). The idea behind the EEGM approach is to distinguish exposure distributions by their mean, which may vary across ancestry groups, *S*.Though *S* is not known, it is reflected in the principal components, *M*, computed from genetic marker data available on all ancestry groups in these data. The expectation of *E* given *M* can be estimated by linear regression of *E* on *M* when *E* is continuous, or by logistic regression when *E* is binary, as in the current study. Thus, for EEGM adjustment, we estimate the expected exposure within ancestral groups with ${\hat{X}}_{E}=\hat{E\left(E|M\right)}$ in the risk model shown in Equation (2) below. We consider EEGM adjustment to be the gold-standard, because, for the case of two ancestral groups, Shin et al. (2012) showed the resulting tests of *G* × *E* interaction achieve the nominal type I error rates. To align this EEGM adjustment results with those based on SRA adjustment, we transform EEGM to the unit interval by subtracting the minimum value and dividing by the range. The use of SRA as a surrogate for *X*_*E*_ is straightforward and leads to more interpretable models (see below), but is subject to some bias, because self-reported ancestry may not accurately reflect genetic ancestry (Wang et al., 2010). To simplify notation, we assume only two self-reported ancestry groups, encoded in ${\hat{X}}_{E}$ as zero or one.

**Figure 1:**
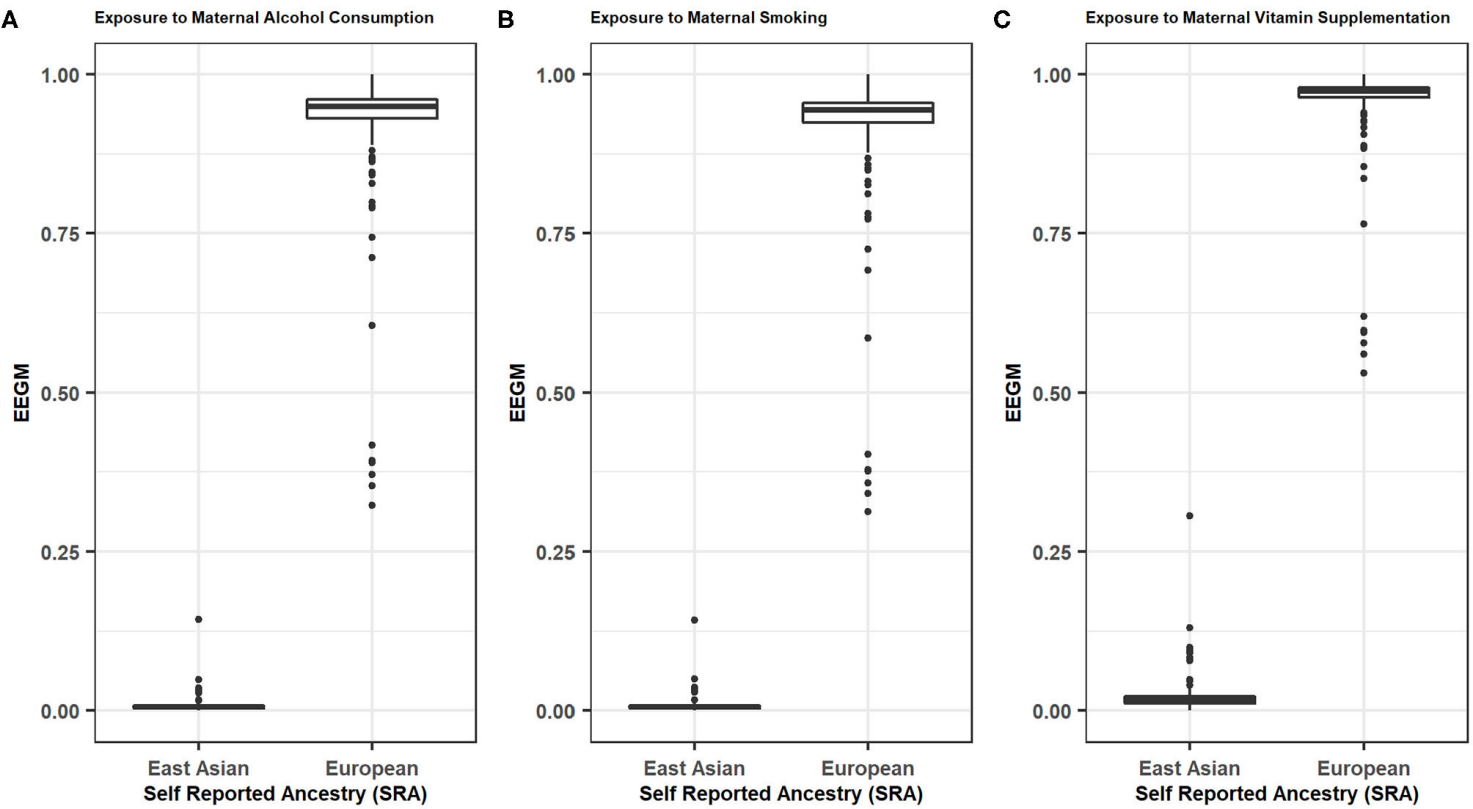
EEGM by SRA for **(A)** Maternal Alcohol Consumptions, **(B)** Maternal Smoking, **(C)** Maternal Vitamin Supplementations.

Distributions of EEGM by SRA for maternal exposure to alcohol, smoking, and vitamin supplements, are shown in Figure 1. As expected, there is a clear separation of EEGM between self-reported Europeans and East Asians for all three exposures considered here. In our analyses we would therefore expect similar results with adjustment by either EEGM or SRA.

For either surrogate ${\hat{X}}_{E}$, let $\beta_{G{\hat{X}}_{E}}$ denote its coefficient so we can write the risk model as

(2) $$\begin{array}{l} \text{log}[P(D=1|G=g,E=e,{\hat{X}}_{E}=x)]=\beta_{0}+g\beta_{G}+e\beta_{E}+x\beta_{{\hat{X}}_{E}}\\ \;+ge\beta_{GE}+gx\beta_{G{\hat{X}}_{E}}+ex\beta_{E{\hat{X}}_{E}}+gex\beta_{GE{\hat{X}}_{E}}, \end{array}$$

leading to genotypic odds

(3) $$\begin{array}{l} \frac{P\left(G=g|D=1,E=e,{\hat{X}}_{E}=x,G_{p}=g_{p}\right)}{P\left(G=g-1|D=1,E=e,{\hat{X}}_{E}=x,G_{p}=g_{p}\right)}\\ =\exp\left(k_{p}+\beta_{G}+e\beta_{GE}+x\beta_{G{\hat{X}}_{E}}+ex\beta_{GE{\hat{X}}_{E}}\right). \end{array}$$

Interpretation of the model in Equation (2) is simplest when ${\hat{X}}_{E}$ is the SRA. If ${\hat{X}}_{E}=0$ for East Asians and 1 for Europeans, then the relative risk of cleft palate due to exposure in self-reported East Asians with *G* = *g* is $e^{\beta_{E}+g\beta_{GE}}$. Thus, $e^{\beta_{GE}}$ is the multiplicative increase in the relative-risk due to exposure for each additional copy of *G* in self-reported East Asians. Similarly, the relative risk of cleft palate due to exposure in self-reported Europeans is $e^{\beta_{E}+\beta_{E\hat{X_{E}}}+g\left(\beta_{GE}+\beta_{GE{\hat{X}}_{E}}\right)}$, so this latter term $e^{\beta_{GE}+\beta_{GE{\hat{X}}_{E}}}$ is the multiplicative increase in the relative-risk due to exposure for each copy of *G* in self-reported Europeans. To summarize, $e^{\beta_{GE}}$ reflects *G* × *E* in self-reported East Asians and $e^{\beta_{GE}+\beta_{GE{\hat{X}}_{E}}}$ reflects *G* × *E* in self-reported Europeans.

Inference can be made from the conditional likelihood based on *P*(*G*|*D* = 1, *E, G*_*p*_), which can be obtained from the genotypic odds shown in Equation (3). Parameter estimates are obtained by maximizing the conditional likelihood, and hypothesis tests are obtained from likelihood ratio statistics. In particular, the hypothesis *H*_*O*_ (i.e., not GxE interaction)$:\beta_{GE}=\beta_{GE{\hat{X}}_{E}}=0$, is tested first by fitting models with and without the interaction terms and comparing the resulting likelihood ratio statistic to the χ^2^ distribution with 2 degrees of freedom.

## 4. Results

Inference was obtained under the unadjusted risk model in Equation (1) and the adjusted risk model in Equation (2) with either SRA or EEGM adjustment. The first analysis was restricted to the 6 SNPs in the *MLLT3* gene reported by Beaty et al. to have significant *G* × *E* with maternal alcohol consumption. *P*-values for the 1 df (no adjustment) and 2 df tests (with ${\hat{X}}_{E}$ adjustment) are shown in Table 3. We see the results with adjustment roughly agree and are only slightly attenuated compared to the results without adjustment for population stratification of exposures.

**Table 3:** *P*-values of 1 df likelihood ratio test from Model (1), and 2 df likelihood ratio tests from models with SRA and EEGM adjustment for the 6 SNPs in the *MLLT3* gene on chromosome 9 show significant evidence of interaction with Maternal Alcohol Consumption.

	**Model 1: 1df LRT**	**Model 2: 2df LRT**	**Model 2: 2df LRT**
**SNP**	**No adj**	**SRA adj**.	**EEGM adj**.
rs4621895	0.0010	0.0043	0.0060
rs4977433	0.0006	0.0035	0.0046
rs6475464	0.0159	0.0128	0.0161
rs668703	0.0014	0.0029	0.0039
rs623828	0.0432	0.1105	0.1358
rs2780841	0.0475	0.1311	0.1683

**Figure 2:**
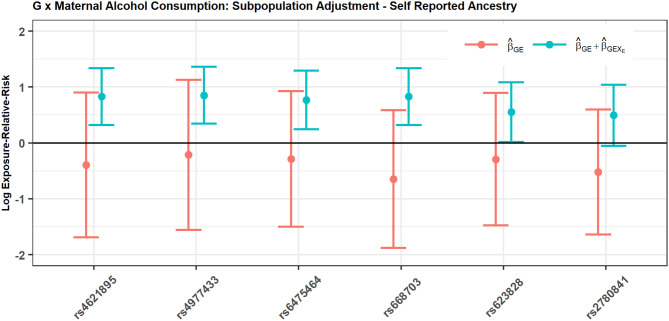
SNPs in *MLLT3* gene show significant interaction with Maternal Alcohol Consumption among European CP trios, but not among East Asian CP trios.

Figure 2 shows the estimated interaction terms ${\hat{\beta }}_{GE}$ and ${\hat{\beta }}_{GE}+{\hat{\beta }}_{GE{\hat{X}}_{E}}$ and their corresponding confidence intervals while Table 4 shows exponentiated parameter estimates and their confidence intervals, as well as *p*-values from inference based on the risk model adjusted by SRA only. The overlap of the confidence intervals for ${\hat{\beta }}_{GE}$ with zero and the non-overlap of the confidence intervals for ${\hat{\beta }}_{GE}+{\hat{\beta }}_{GE{\hat{X}}_{E}}$ suggest no evidence of *G* × *E* interaction in self-reported East Asians, but evidence of *G* × *E* interaction in self-reported Europeans. The table of exponentiated parameter estimates quantifies this apparent *G* × *E* in self-reported Europeans. For example, the estimate $e^{{\hat{\beta }}_{GE}+{\hat{\beta }}_{GE{\hat{X}}_{E}}}=2.2918$ suggests that each copy of the minor allele at SNP rs4621895 more than doubles the relative risk of CP due to maternal alcohol exposure.

**Table 4:** Exponentiated *G* × *E* parameter estimates with corresponding exponentiated 95% Confidence Intervals with SRA adjustment at 6 the SNPs on *MLLT3* (Chr 9) showing significant interaction with Maternal Alcohol Consumption.

**SNP**	**$e^{{\hat{\beta }}_{GE}}$**	**95% CI**	**$e^{{\hat{\beta }}_{GE}+{\hat{\beta }}_{GE{\hat{X}}_{E}}}$**	**95% CI**	***P*-value**
rs4621895	0.6732	(0.1845, 2.4563)	2.2918	(1.3771, 3.8143)	0.0043
rs4977433	0.8078	(0.2109, 3.0943)	2.3484	(1.4121, 3.9054)	0.0035
rs6475464	0.7507	(0.2228, 2.5296)	2.1621	(1.2792, 3.6545)	0.0128
rs668703	0.5243	(0.1532, 1.7943)	2.2913	(1.3786, 3.8082)	0.0029
rs623828	0.7475	(0.2285, 2.4449)	1.7385	(1.0194, 2.9649)	0.1105
rs2780841	0.5946	(0.1941, 1.8215)	1.6388	(0.9510, 2.8241)	0.1311

Beaty et al. report that CP-affected European trios were gathered from multiple sites. Given that the *G* × *E* signal for SNPs in *MLLT3* appears to be driven by these trios of European ancestry only, we also investigated whether stratification within Europeans could explain the apparent *G* × *E* interaction. To do this, we computed principal components from genetic markers in all self-reported European trios and performed logistic regression of exposure on PCs computed from 6,231,573 SNPs. However, the PCs were not predictive of exposure, and so there was no evidence of ancestral sub-groups with different *E* distributions within self-reported Europeans.

We performed similar analyses to the other four genes implicated in the Beaty study with mixed success. In some instances, the SNPs and exposures were so rare in the self-reported East Asian trios the $\beta_{GE{\hat{X}}_{E}}$ coefficient in the model (2) could not be estimated. In such situations, we must assume a common *G* × *E* effect in both East Asians and Europeans. However, in this extended analysis of the other 4 genes we were still able to find some evidence of *G* × *E* interaction, though, as in Table 3, the results were attenuated compared to those when there is no adjustment for confounding. Tables of results are shown in Appendix A.2.

## 5. Discussion and Conclusions

Case-parent trio studies allow robust inference of genetic main effects, but inference of *G* × *E* interaction is susceptible to bias from a particular form of confounding known as exposure-related population structure. In the GENEVA Oral Cleft Study, such confounding would arise from heterogeneity of haplotype and exposure distributions across component sub-populations of the study. This is a concern because of the observed heterogeneity in exposure to maternal tobacco, alcohol, and multivitamin use among European and East Asian populations. We emphasize that differences in exposure need not be genetic in nature; indeed, the differences in exposure distributions in the GENEVA Study are presumably cultural. However, if these non-genetic differences coincide with genetic differences in genes such as *MLLT3, SMC2, TBK1, ZNF236*, and *BAALC*, they can result in spurious inference of *G* × *E* interaction. We applied the bias-reduced methods of Shin et al. to the GENEVA Oral Cleft Study and generally obtained the same conclusions regarding *G* × *E* interaction as an earlier analysis by Beaty et al. However, we found that the data only supported genetic modifiers of the exposure effects in self-reported Europeans. By contrast, all three exposures are too rare in self-reported East Asians to draw any conclusions about the presence of such modifiers in that population.

We further investigated whether confounding within the self-reported Europeans could explain these suggestions of *G* × *E* interaction. However, the genetic marker data from the genome-wide marker panel was not predictive of exposure within this population and so the adjustment of Shin et al. was not possible. Along the same lines, we investigated whether confounding in self-reported East Asians could explain the *G* × *E* findings of Wu et al., who found evidence of genetic modifiers of the effect of environmental tobacco exposure on CP. The study sample was comprised of trios from Korea, China (Hubei, Sichuan, and Shandong provinces), Taiwan, and Singapore. Differences in exposure rates and haplotype distributions among these populations could lead to spurious evidence of *G* × *E* interaction. Again, we found genetic markers were not predictive of exposure, so the methods of Shin et al. could not be applied directly. Both of these scenarios suggest the need for alternative methods for adjusting for exposure-related stratification among sub-populations.

**Figure 3:**
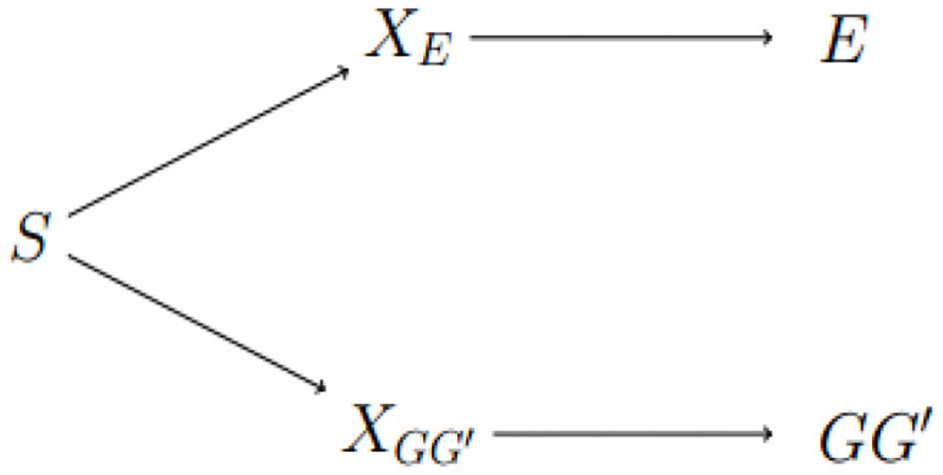
Dependence between *E* and *GG*′ is through latent sub-population S. Latent factors *X*_*E*_ and $X_{GG^{\prime }}$ indicate different distributions of *E* and *GG*′, respectively. *E* and *GG*′ are conditionally independent given any of the three variables on the path between them.

We conclude with a discussion of areas for future work. Exposure-related population structure is illustrated in Figure 3. The link between *GG*′ haplotype frequencies and *E* distributions can be broken by adding any of *S*, *X*_*E*_, or $X_{GG}^{\prime }$to the risk model. The approach of Shin et al. is to estimate a surrogate for *X*_*E*_. As noted above, this approach may not be sensitive enough to adjust for subtle heterogeneity in genetic and exposure distributions. The diagram suggests alternatives, namely conditioning on *S* or on $X_{GG^{\prime }}$. The advantage of conditioning on *S*, or on principal components that reflect *S*, is its simplicity and familiarity to researchers who study unrelated subjects. The potential advantage of an approach based on inferring $X_{GG^{\prime }}$ is that it aims to characterize local structure in the genome of study subjects that could be responsible for apparent differences in risk at the test locus caused by differences in Linkage Disequilibrium (LD) with a nearby causal locus. Efforts to develop such methods are ongoing.

Another possible avenue for future research is to develop an approach that is a hybrid of case-parent-trio and case-only designs. The case-only design (Piegorsch et al., 1994) shares key features with the case-parent trio design. Starting with a log-linear model of disease probabilities, and assuming *G* and *E* are independent, *G* × *E* is inferred from an association between *G* and *E* in cases. By conditioning on parental genotypes, inference from case-parent trio data is under the weaker assumption that *G* and *E* are independent within *families*, rather than in the general population, as in the case-only design. However, the number of cases available for analysis may be increased by dropping the requirement of parental genotype data. Thus, robust methods for inference of *G* × *E* that make use of cases with or without parental genotypes become of interest.

## Data Availability Statement

Publicly available datasets were analyzed in this study. This data can be found at: https://www.ncbi.nlm.nih.gov/projects/gap/cgi-bin/study.cgi?study_id=phs000094.v1.p1 (dbGaP Study Accession: phs000094.v1.p1).

## Author Contributions

PR researched the statistical methods, carried out the analyses, and wrote the manuscript. BM developed the research question, consulted on the analyses, and wrote and proofread the manuscript. All authors contributed to the article and approved the submitted version.

## Conflict of Interest

The authors declare that the research was conducted in the absence of any commercial or financial relationships that could be construed as a potential conflict of interest.
