# Supplementary material for: Re-analysis of a Genome-Wide Gene-By-Environment Interaction Study of Case Parent Trios, Adjusted for Population Stratification
Source: Front Genet. 2021 Jan 13;11:600232. doi: 10.3389/fgene.2020.600232 (PMC7838675; doi:10.3389/fgene.2020.600232)
Supplement: Supplementary file 1 [file Data_Sheet_1.pdf]

frontiers

Supplementary Material

A APPENDIX

A.1 Genotypic Odds

Assuming G and E are conditionally independent given parental genotypes G_p, and conditioning on G_p and E we can obtain a likelihood (Shin et al. [2012]),

$$P(G = g \mid D = 1, E = e, G_p = g_p) = \frac{P(D = 1 \mid G = g, E = e)P(G = g \mid G_p = g_p)}{\sum_{g^*} P(D = 1 \mid G = g^*, E = e)P(G = g^* \mid G_p = g_p)}$$

We could use this likelihood to define Genotypic Odds,

$$GO_g(e) = \frac{P(G = g \mid D = 1, E = e, G_p = g_p)}{P(G = g - 1 \mid D = 1, E = e, G_p = g_p)} = \frac{P(D = 1 \mid G = g, E = e)}{P(D = 1 \mid G = g - 1, E = e)} \times \frac{P(G = g \mid G_p = g_p)}{P(G = g - 1 \mid G_p = g_p)}$$

$$GO_g(e) = \frac{P(G = g \mid D = 1, E = e, G_p = g_p)}{P(G = g - 1 \mid D = 1, E = e, G_p = g_p)} = exp\{\beta_G + e\beta_{GE}\} \times exp\{\kappa_p\}$$

where $\kappa_p = ln(2)$ if $g = 1$ and $\kappa_p = -ln(2)$ if $g = 2$ given that parental genotypes, G_p, are both heterozygous.

A.2 $G \times E$ parameter estimates and its 95% confidence intervals

We have listed the sub-population specific $G \times E$ interaction parameter estimates and their corresponding 95% confidence intervals with the EEGM adjustment in Tables A1 to A5. These five tables correspond to the five genes which Beaty *et al.* found evidence for $G \times E$ for maternal alcohol consumption and SNPs in the genes *MLLT3* and *SMC2*, for maternal smoking and SNPs in the genes *TBK1* and *ZNF236*, and for maternal multivitamin use and SNPs in the *BAALC* gene respectively. At four SNPs shown in Table A2 and at a one SNP of Table A3, the exposures were so rare in the self-reported East Asian trios the $\beta_{GE\hat{X}_E}$ coefficient in the model (2) could not be estimated. In such situations, we assumed a common $G \times E$ effect in both East Asians and Europeans.

SNP	$e^{\hat{\beta}_{GE}}$	95% CI	$e^{\hat{\beta}_{GE}+\hat{\beta}_{GE\hat{X}_E}}$	95% CI	P-value
rs4621895	0.7430	(0.2038, 2.7084)	2.4089	(1.3935, 4.1640)	0.0060
rs4977433	0.9004	(0.2339, 3.4659)	2.4451	(1.4156, 4.2234)	0.0046
rs6475464	0.8351	(0.2468, 2.8256)	2.2750	(1.2893, 4.0141)	0.0161
rs668703	0.5679	(0.1674, 1.9268)	2.4667	(1.4288, 4.2587)	0.0039
rs623828	0.7785	(0.2367, 2.5608)	1.7828	(1.0062, 3.1589)	0.1358
rs2780841	0.6244	(0.2044, 1.9072)	1.6936	(0.9451, 3.0348)	0.1683

Table A1. $G \times E$ parameter estimates and its 95% Confidence Intervals obtained with EEGM adjustment of CP children at 6 SNPs on *MLLT3* gene (Chr 9) which showed evidence significant interaction with Maternal Alcohol Consumption.

SNP	$e^{\hat{\beta}_{GE}}$	95% CI	$e^{\hat{\beta}_{GE}+\hat{\beta}_{GE}\hat{X_E}}$	95% CI	P-value
rs10125685	4.2540	(1.4359, 12.6025)	-	-	0.0056
rs628345	2.1111	(0.9776, 4.5590)	-	-	0.0533
rs630103	1.6991	(0.4002, 7.2138)	0.9415	(0.5505, 1.6100)	0.7645
rs868619	6.0936	(0.2122, 174.9883)	1.1553	(0.6562, 2.0342)	0.3139
rs1536895	5.0690	(1.6175, 15.8859)	-	-	0.0026
rs10217601	2.1076	(0.9758, 4.5523)	-	-	0.0538

Table A2. $G \times E$ parameter estimates and its 95% Confidence Intervals obtained with EEGM adjustment of CP children at 6 SNPs on *SMC2* gene (Chr 9) which showed evidence of significant interaction with Maternal Alcohol Consumption.

SNP	$e^{\hat{\beta}_{GE}}$	95% CI	$e^{\hat{\beta}_{GE}+\hat{\beta}_{GE}\hat{X_E}}$	95% CI	P-value
rs1317532	1.3652	(0.2971, 6.2738)	1.5768	(0.8682, 2.8636)	0.2522
rs1317535	1.9270	(0.3337, 11.1292)	1.4510	(0.7517, 2.8011)	0.3361
rs2141765	1.7960	(0.3326, 9.6983)	1.7137	(0.9524, 3.0836)	0.1071
rs7969932	2.1652	(0.2249, 20.8446)	2.0355	(1.0992, 3.7693)	0.0294
rs6581575	2.2341	(0.2336, 21.3651)	2.0304	(1.0964, 3.7598)	0.0289
rs4964110	1.7773	(0.1444, 21.8819)	1.3237	(0.4052, 4.3244)	0.7707
rs10506538	1.8945	(0.6335, 5.6655)	-	-	0.2470
rs4964090	1.8842	(0.4499, 7.8906)	2.0321	(1.0955, 3.7696)	0.0322
rs7963840	1.2831	(0.2771, 5.9417)	1.7063	(0.9226, 3.1556)	0.1867

Table A3. $G \times E$ parameter estimates and its 95% Confidence Intervals obtained with EEGM adjustment of CP children at 9 SNPs on *TBK1* gene (Chr 12) which showed evidence of significant interaction with Maternal Smoking.

SNP	$e^{\hat{\beta}_{GE}}$	95% CI	$e^{\hat{\beta}_{GE}+\hat{\beta}_{GEX}\hat{x}_E}$	95% CI	P-value
rs8091823	0.6904	(0.1239, 3.8469)	2.2546	(1.1529, 4.4088)	0.0540
rs3752075	2.0202	(0.3139, 13.0032)	2.2164	(1.2009, 4.0906)	0.0144
rs9960774	0.5322	(0.1020, 2.7769)	2.3881	(1.2061, 4.7284)	0.0365
rs486131	0.7675	(0.0480, 12.2659)	1.6425	(0.8236, 3.2756)	0.3583
rs10469070	1.3173	(0.0804, 21.5748)	2.2320	(0.6725, 7.4082)	0.3704
rs470385	0.1785	(0.0217, 1.4702)	1.6756	(0.6281, 4.4697)	0.1191
rs470560	0.5898	(0.1141, 3.0499)	2.7249	(1.4292, 5.1950)	0.0075
rs470563	0.5861	(0.1133, 3.0307)	2.9330	(1.5288, 5.6270)	0.0037
rs470337	1.3210	(0.1235, 14.1317)	0.7956	(0.3298, 1.9195)	0.8692
rs8095808	0.5681	(0.1027, 3.1413)	2.3192	(1.2088, 4.4493)	0.0350

Table A4. $G \times E$ parameter estimates and its 95% Confidence Intervals obtained with EEGM adjustment of CP children at 10 SNPs on *ZNF236* gene (Chr 18) which showed evidence of significant interaction with Maternal Smoking.

SNP	$e^{\hat{\beta}_{GE}}$	95% CI	$e^{\hat{\beta}_{GE}+\hat{\beta}_{GEX}\hat{x}_E}$	95% CI	P-value
rs963599	1.5394	(0.7968, 2.9741)	1.6378	(0.9228, 2.9068)	0.0891
rs10955309	2.3263	(1.0711, 5.0523)	2.8767	(1.3475, 6.1415)	0.0013
rs1473541	1.3775	(0.7534, 2.5187)	1.2450	(0.6712, 2.3091)	0.4343
rs7814399	1.8379	(0.9504, 3.5543)	1.1470	(0.6365, 2.0669)	0.1590
rs3736042	1.8110	(0.9221, 3.5569)	2.1372	(1.0169, 4.4917)	0.0222
rs2454013	1.2588	(0.6985, 2.2685)	1.7823	(1.0409, 3.0520)	0.0710
rs2935579	1.2062	(0.6617, 2.1986)	1.2443	(0.6398, 2.4201)	0.6568
rs1874091	1.1328	(0.4507, 2.8471)	0.8685	(0.4553, 1.6566)	0.8874
rs1845430	1.3354	(0.7365, 2.4213)	1.7155	(0.9658, 3.0470)	0.1021
rs6468861	1.2080	(0.6531, 2.2345)	1.6268	(0.9390, 2.8185)	0.1699
rs6468862	1.6857	(0.2667, 10.6563)	1.6463	(0.9364, 2.8941)	0.1636

Table A5. $G \times E$ parameter estimates and its 95% Confidence Intervals obtained with EEGM adjustment of CP children at 11 SNPs on *BAALC* gene (Chr 8) which showed evidence of significant interaction with Maternal Vitamin Supplementation.

REFERENCES

J.-H. Shin, C. Infante-Rivard, J. Graham, and B. McNeney. Adjusting for Spurious Gene-by-Environment Interaction Using Case-Parent Triads. *Statistical Applications in Genetics and Molecular Biology*, 11(2), jan 2012. doi: 10.2202/1544-6115.1714. URL `https://doi.org/10.2202%2F1544-6115.1714`.
